# Vitamin D_2_ Supplementation Amplifies Eccentric Exercise-Induced Muscle Damage in NASCAR Pit Crew Athletes

**DOI:** 10.3390/nu6010063

**Published:** 2013-12-20

**Authors:** David C. Nieman, Nicholas D. Gillitt, R. Andrew Shanely, Dustin Dew, Mary Pat Meaney, Beibei Luo

**Affiliations:** 1 Human Performance Laboratory, Appalachian State University, North Carolina Research Campus, Kannapolis, NC 28081, USA; E-Mails: shanelyra@appstate.edu (R.A.S.); dustindew@gmail.com (D.D.); meaneymp@appstate.edu (M.P.M.); 2 Dole Nutrition Research Laboratory, North Carolina Research Campus, Kannapolis, NC 28081, USA; E-Mail: Nicholas.Gillitt@dole.com; 3 Key Laboratory of Exercise and Health Sciences of Ministry of Education, Shanghai University of Sport, Shanghai 200438, China; E-Mail: lbb0220@126.com

**Keywords:** delayed onset of muscle soreness (DOMS), myoglobin, creatine phosphokinase, muscle function testing, eccentric exercise, LC-MS/MS

## Abstract

This study determined if 6-weeks vitamin D_2_ supplementation (vitD_2_, 3800 IU/day) had an influence on muscle function, eccentric exercise-induced muscle damage (EIMD), and delayed onset of muscle soreness (DOMS) in National Association for Stock Car Auto Racing (NASCAR) NASCAR pit crew athletes. Subjects were randomized to vitD_2_ (*n* = 13) and placebo (*n* = 15), and ingested supplements (double-blind) for six weeks. Blood samples were collected and muscle function tests conducted pre- and post-study (leg-back and hand grip dynamometer strength tests, body weight bench press to exhaustion, vertical jump, 30-s Wingate test). Post-study, subjects engaged in 90 min eccentric-based exercise, with blood samples and DOMS ratings obtained immediately after and 1- and 2-days post-exercise. Six weeks vitD_2_ increased serum 25(OH)D_2_ 456% and decreased 25(OH)D_3_ 21% *versus* placebo (*p* < 0.001, *p* = 0.036, respectively), with no influence on muscle function test scores. The post-study eccentric exercise bout induced EIMD and DOMS, with higher muscle damage biomarkers measured in vitD_2_ compared to placebo (myoglobin 252%, 122% increase, respectively, *p* = 0.001; creatine phosphokinase 24 h post-exercise, 169%, 32%, *p* < 0.001), with no differences for DOMS. In summary, 6-weeks vitD_2_ (3800 IU/day) significantly increased 25(OH)D_2_ and decreased 25(OH)D_3_, had no effect on muscle function tests, and amplified muscle damage markers in NASCAR pit crew athletes following eccentric exercise.

## 1. Introduction

Vitamin D deficiency is defined as a serum 25-hydroxyvitamin D (25(OH)D) concentration of 20 ng/mL or less, with vitamin D insufficiency established as 21–29 ng/mL [1]. Recent evidence suggests that optimal vitamin D status, defined by estimated maximum parathyroid (PTH) suppression, occurs at 25(OH)D levels of 40 ng/mL and higher [2,3]. Estimates from the National Health and Nutrition Examination Survey (NHANES) are that three in four individuals in the U.S. population have 25(OH)D levels less than 30 ng/mL [3].

A high proportion of athletes are also vitamin D insufficient, with prevalence rates varying according to sun exposure, time of the year, and residential latitude [4,5,6,7,8]. Early 20th century studies suggested that ultraviolet (UV) irradiation improved physical performance, and that physical training responses peaked in late summer [4]. More recent studies report that vitamin D receptors (VDR) are present in skeletal muscle, and that vitamin D treatment of deficient individuals improves muscular strength and Type II muscle fiber size [2,9,10,11]. Epidemiologic studies of elderly individuals support direct associations between 25(OH)D levels and physical performance, with some support in randomized clinical trials, especially among vitamin D deficient adults [12,13,14,15,16]. A few epidemiologic studies support relationships between 25(OH)D and performance across all ages in adults [17,18,19].

Limited evidence suggests that treatment of vitamin D insufficient athletes may improve performance [20,21]. In the UK, 5000 IU/day vitamin D_3_ supplementation for eight weeks improved 10 m sprint times and vertical jump performance in athletes who started the study with a mean serum 25(OH)D level of 12 ng/mL [5]. Maintaining adequate vitamin D status may also reduce inflammation and aid in recovery from injury or intensive workouts, but data in humans are inconsistent [4,17]. One study showed that vitamin D_3_-treated rats experienced attenuation in plasma creatine kinase (CK) and inflammation biomarkers following high-intensity exercise, with an increase in muscle VDR protein expression [22]. No previous human study has been published regarding the effect of vitamin D supplementation in countering eccentric exercise-induced muscle damage (EIMD) and delayed onset of muscle soreness (DOMS). We hypothesized that 6-weeks supplementation with vitamin D (3800 IU/day) using vitamin D_2_ Portobello mushroom powder would improve muscle function and strength, and attenuate EIMD and DOMS in NASCAR pit crew athletes during their off-season in December and January.

## 2. Experimental Section

### 2.1. Subjects

NASCAR pit crew athletes (*n* = 30) from Hendrick Motorsports (Concord, North Carolina, NC, USA) were recruited and invited to join the study if they agreed to avoid: (1) food and supplement sources (during the 6-week supplementation period) that were high in vitamin D (specifically canned fish, cod liver oil, salmon, and supplements with high-dose vitamin D); (2) large dose vitamin/mineral supplements (above 100% recommended dietary allowances); (3) anti-inflammatory medications; (4) tanning beds and prolonged sun exposure. The Appalachian State University institutional review board approved all experimental procedures.

### 2.2. Research Design

Pit crew members provided blood samples in mid-October (fall baseline for serum vitamin D status) and then again during baseline testing (first week of December). Baseline testing consisted of the leg-back dynamometer strength test, hand-grip dynamometer strength test, body weight bench press to exhaustion, vertical jump, and 30-s Wingate anaerobic power cycling test. Height, weight, and percent body fat (three skinfolds) were also obtained.

1 Leg-back dynamometer strength test: With arms straight and knees slightly bent, subjects grasped a bar that was attached to a platform via a chain and dynamometer (Lafayette Instruments, Lafayette, IN, USA), and then lifted up with maximal effort for several seconds. The test was repeated three times, with the highest score recorded;
2 Hand-grip dynamometer strength test: The hand-grip dynamometer (Lafayette Instruments, Lafayette, IN, USA) was adjusted to hand size (with the middle of the fingers on the handle). The subject assumed a slightly bent forward position with the right hand hanging down and forward, and then gripped maximally for 2–3 s. The best of three trials was recorded;
3 Body weight bench press to exhaustion: Subjects bench pressed a weighted bar equal to body weight as many times as possible (to a metronome set at 60 beats/min or 30 lifts/min) until fatigue. The bar touched a small foam block on the chest lightly in the down position, and lifted upwards until the arms were straight in the up position;
4 Vertical jump: Subjects first stood erect with the feet flat on the floor and reached as high as possible with both arms and hands (standing reach height). Subjects then squatted down and jumped as high as possible with one arm and hand, and tapped the measuring device (jump height) (Vertec vertical jump apparatus, Questtek Corp, Northridge, CA, USA). This was repeated three times, with the best score recorded as the difference between the jump and standing reach heights;
5 Wingate anaerobic power cycling test: The Lode cycle ergometer (Lode B.V., Groningen, The Netherlands) was adjusted to the body mass of the subject (7 W per kilogram), and then subjects cycled at maximal speed for 30 s. The peak and total wattage power output was recorded and adjusted to body mass.

Subjects were randomized to vitamin D or placebo groups, and ingested the supplement for six weeks. Following supplementation, blood samples were collected and subjects repeated the muscle function tests. Subjects then engaged in 90 min of eccentric-based exercise. Blood samples were obtained immediately following exercise, and then 24-h- and 48-h-post-exercise. The blood samples were analyzed for serum vitamin D and muscle damage biomarkers. DOMS was measured using a Likert-scale questionnaire [23] pre- and post-supplementation, and immediately post- and 24-h and 48-h after the 90 min of eccentric exercise bout.

### 2.3. Eccentric Exercise

The 90 min eccentric exercise bout consisted of 17 different exercises:

(1) Hammer incline presses with eccentric focus (3 sets, 5 reps); (2) bench presses with resistance bands (3 sets, 20 s to fatigue); (3) supine medicine ball (9.1 kg) explosive catch and throws (20 s, 3 sets); (4) bent (90°) arm hangs to fatigue (3 sets); (5) eccentric lat pulls (8 reps, 3 sets); (6) partner rowing eccentric pulls (8 reps per arm, 3 sets); (7) eccentric triceps extensions (8 reps per arm, 3 sets); (8) eccentric bicep curls (8 reps, 3 sets); (9) explosive tuck jumps (20 s, 3 sets); (10) eccentric back extensions (8 reps, 3 sets); (11) eccentric hamstring curls (8 reps, 3 sets); (12) 20 s sprints on inclined treadmills with no electrical power (3 sets); (13) split squats (15 reps each leg, 2 sets); (14) walk 0.40 km with dumbbells and shoulder shrug every three steps; (15) isometric abdominal curl with medicine ball (9.1 kg) twisting side to side for 20 s (3 sets); (16) abdominal crunches for 20 s (3 sets); (17) plank position (elbows and toes) for 45 s (2 sets).

### 2.4. Supplement

Fresh Portobello mushrooms (*Agaricus bisporus*) were air dried at 71–76 °C for 48–72 h in a convection dryer [24]. Once dried, the mushrooms pieces were milled to approximately 35 mesh, or 500 µm. The powder was then treated on a vibrating conveyor with pulsed light (UVB) from a Xenon broad spectrum lamp (100–800 nm) operating at 3 pulses per second for a total of thirty 2 ms pulses which converted ergosterol to ergocalciferol (vitamin D_2_). Subjects were given Portobello mushroom powder with or without vitamin D_2_ mixed in soymilk powder (non-vitamin D fortified) in six plastic containers (one for each week of the study). Subjects ingested one level teaspoon of the product each day (with or without 3800 IU vitamin D_2_) during breakfast.

### 2.5. Mushroom Vitamin D_2_ Supplement Analysis

The mushroom vitamin D_2_ analysis has been described fully [24,25]. Briefly, mushroom powder samples underwent 3 h saponification at room temperature. The final extract was dried and reconstituted in absolute ethanol for LC-MS/MS analysis. Vitamin D_2_ was quantitatively determined through comparison to internal standard responses. An Accela HPLC system coupled with a PDA and a LTQ Velos tandem mass spectrometer system (Thermo Scientific) was used for liquid chromatographic separation and quantitation of vitamin D_2_ in samples.

### 2.6. Analytical Measures

Blood samples were drawn from the antecubital vein by standard venipuncture by a trained phlebotomist. All samples were drawn into vacuum blood collection tubes without additives, allowed to coagulate for 20 min at room temperature, and centrifuged. Serum myoglobin was measured with the Elecsys Myoglobin electrochemiluminescence immunoassay kit (Roche Diagnostics, Indianapolis, IN, USA) using the Modular Analytics E170 (Roche Diagnostics, Indianapolis, IN, USA). The sensitivity of the myoglobin assay is 1 ng/mL and the coefficient of inter-assay variation is 3.1%. Serum samples were individually assessed for lactate dehydrogenase (LDH) and creatine phosphokinase (CK) with reagent specific enzymatic assays using the SYNCHRON LX^®^ System (Beckman Coulter, Brea, CA, USA). The sensitivity of the LDH and CK assays was 5 IU/L and the coefficient of inter-assay variation was 5.3% and 4.5%, respectively.

Analysis of serum 25-hydroxyvitamin D_2_ and D_3_ was measured by HPLC-MS/MS, as previously described [24]. Serum samples as well as calibration standards, water blanks, serum blanks and QCs were prepared as previously described [26], and analyzed on the same LC-MS system described above.

### 2.7. Statistics

Data are expressed as mean ± SE. Subject characteristics were compared between groups using independent *t*-tests. Muscle function, serum vitamin D, DOMS, and muscle damage data were analyzed using 2 (group) × 2 to 5 (time) repeated-measures ANOVAs. When interaction effects were significant (*p* ≤ 0.05), changes between time points within groups were compared across time points using independent *t*-tests.

## 3. Results

Subject characteristics for the placebo (*n* = 15) and vitamin D (*n* = 13) groups are compared in Table 1, with no differences noted for age, height, body mass, and body composition.

**Table 1:** Subject characteristics.

Variable	Placebo (*n* = 15)	Vitamin D (*n* = 13)	*p*-Value
Age (years)	27.3 ± 0.9	27.1 ± 1.5	0.880
Height (m)	1.86 ± 0.02	1.85 ± 0.02	0.792
Body mass (kg)	97.7 ± 3.7	102 ± 5.8	0.486
Body fat (%)	13.8 ± 0.9	14.8 ± 1.5	0.534

**Figure 1:**
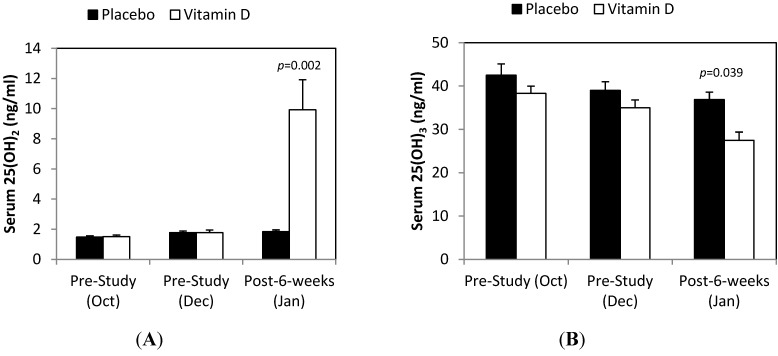
Serum levels for vitamin D_2_ (**A**) and D_3_ (**B**) pre-study (October and December), and after 6 weeks supplementation with mushroom vitamin D_2_ or placebo. Interaction effects, *p* = 0.001 for vitamin D_2_ and *p* = 0.023 for vitamin D_3_, and time effects, *p* < 0.001 for both. Chart *p*-value represents contrast at time point.

Total serum 25(OH)D was 43.7 ± 2.7 and 39.6 ± 1.6 ng/mL in the placebo and vitamin D groups, respectively, during October, 40.7 ± 2.1 and 36.6 ± 1.7 ng/mL in December (pre-supplementation), and 38.6 ± 1.8 and 37.4 ± 1.9 ng/mL in January (post-supplementation) (time effect *p* = 0.001, interaction effect *p* = 0.238). Supplementation with mushroom vitamin D_2_ powder for 6 weeks caused no significant change in 25(OH)D (*p* = 0.127), a significant increase in serum 25(OH)D_2_ (8.14 ± 1.96 ng/mL, *p* < 0.001) and a significant decrease in serum 25(OH)D_3_ (−7.48 ± 2.28 ng/mL, *p* = 0.036) compared to placebo (0.076 ± 1.19 ng/mL and −2.11 ± 1.09 ng/mL, respectively) (Figure 1A,B). Serum 25(OH)D_3_ was highest in October and then decreased to levels measured in December and January in the placebo group (within group contrasts, *p* < 0.01).

Pre-to-post-study measurements for muscle function, including leg-back and hand grip dynamometer tests, the body mass bench press test, vertical jump, and the 30 s Wingate test) did not differ between groups (all interaction effects, *p* > 0.05) (Table 2).

**Table 2:** Muscle function tests, pre- and post-6 weeks supplementation.

Variable	Placebo (*N* = 15)	Vitamin D (*N* = 13)	Interaction Effect *p*-Value
Leg-Back Dynamometer (kg)			
Pre-Study	187 ± 6.4	190 ± 6.1	0.133
6-weeks	218 ± 8.6	200 ± 5.7	
Hand Grip Dynamometer (kg)			
Pre-Study	47.4 ± 2.1	50.2 ± 2.0	0.208
6-weeks	48.3 ± 2.4	53.7 ± 2.1	
Bench Press (reps, body mass)			
Pre-Study	17.4 ± 1.6	15.7 ± 1.4	0.083
6-weeks	16.5 ± 1.5	17.3 ± 1.4	
Vertical Jump (inches)			
Pre-Study	29.3 ± 0.6	29.8 ± 1.2	0.286
6-weeks	29.2 ± 0.5	30.4 ± 1.3	
Wingate, Peak Power (W/kg)			
Pre-Study	16.6 ± 0.6	15.7 ± 0.9	0.967
6-weeks	16.7 ± 0.7	15.8 ± 1.1	
Wingate, Anaerobic Capacity (W/kg)			
Pre-Study	9.11 ± 0.2	8.71 ± 0.3	0.723
6-weeks	9.01 ± 0.2	8.56 ± 0.3	

**Figure 2:**
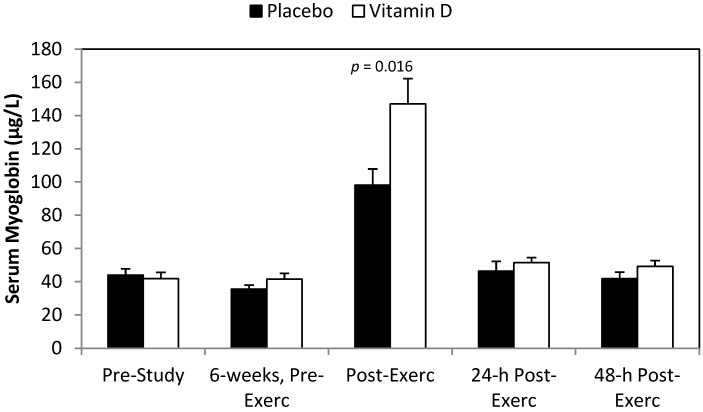
Serum myoglobin before and after 6 weeks supplementation with mushroom vitamin D_2_ or placebo, and immediately post-, 24-h-post-, and 48-h-post-eccentric exercise. Interaction effect, *p* < 0.001, and time effect, *p* < 0.001. Chart *p*-value represents contrast.

**Figure 3:**
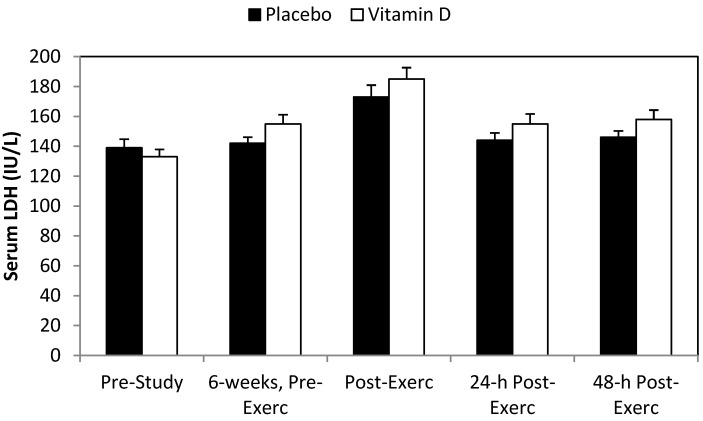
Serum lactate dehydrogenease (LDH) before and after 6 weeks supplementation with mushroom vitamin D_2_ or placebo, and immediately post-, 24-h-post-, and 48-h-post-eccentric exercise. Interaction effect, *p* = 0.065, and time effect, *p* < 0.001.

**Figure 4:**
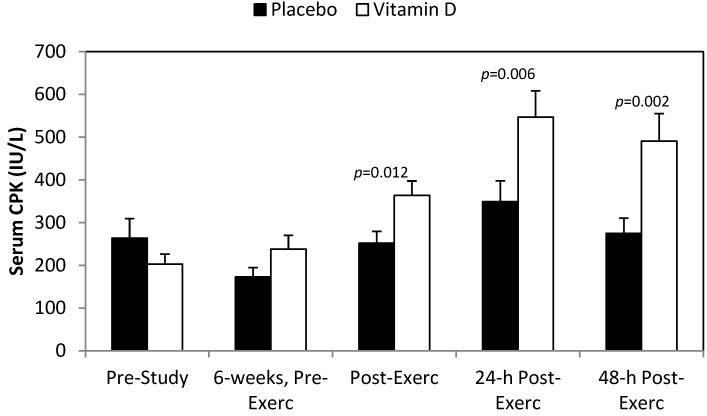
Serum creatine phosphokinase (CK) before and after 6 weeks supplementation with mushroom vitamin D_2_ or placebo, and immediately post-, 24-h-post-, and 48-h-post-eccentric exercise. Interaction effect, *p* < 0.001, and time effect, *p* < 0.001.

**Figure 5:**
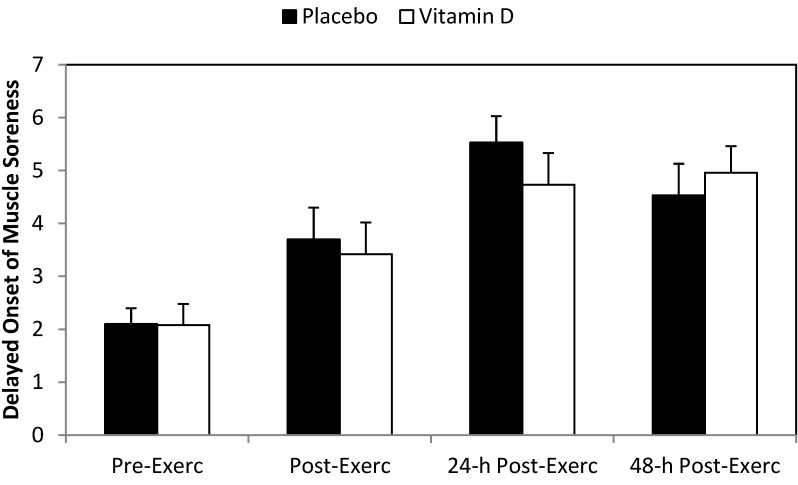
Delayed onset of muscle soreness (DOMS) before and immediately post-, 24-h-post-, and 48-h-post-eccentric exercise. Interaction effect, *p* = 0.490, and time effect, *p* < 0.001.

The post-study eccentric training bout caused significant increases in serum myoglobin (Figure 2), LDH (Figure 3), CK (Figure 4), and DOMS (Figure 5) (all time effects, *p* < 0.001). Significantly higher post-exercise serum levels for myoglobin and CK were measured in the vitamin D_2_ compared to placebo group (both interaction effects, *p* < 0.001), with a trend for higher serum LDH levels (interaction effect, *p* = 0.065). The pattern of change in DOMS did not differ between groups (interaction effect, *p* = 0.490). For the whole group, change in serum 25(OH)D_2_ correlated significantly with change in post-exercise serum myoglobin (*r* = 0.57, *p* = 0.001).

## 4. Discussion

Contrary to our hypothesis, high-dose vitamin D_2_ supplementation in NASCAR pit-crew athletes during a 6-week period in December and January amplified EIMD and had no effect on muscle function. Vitamin D_2_ supplementation increased serum 25(OH)D_2_ ~8 ng/mL but decreased serum 25(OH)D_3_ ~7.5 ng/mL, with no significant change in total 25(OH)D.

These results differ from those of Choi *et al*. [22] who showed that large-dose vitamin D_3_ supplementation in rats (i.p. 1000 IU/kg body weight) countered muscle damage and inflammation induced by high-intensity exercise. Vitamin D_3_-treated rats had highly increased protein expression of VDR in muscles, lower post-exercise levels of plasma CK and LDH, and reduced phosphorylation of AMPK and gene expression for IL-6 and TNF-α compared to controls. A major difference in the current study was the use of mushroom vitamin D_2_ powder.

Aside from oily fish, few foods contain natural vitamin D, and thus most treatments utilize vitamin D supplements containing ergocalciferol (vitamin D_2_) and cholecalciferol (vitamin D_3_). Vitamin D_2_ is the artificial form of vitamin D derived from irradiation of the plant sterol, ergosterol, and is often used in food fortification, dietary supplements, and pharmaceutical preparations. Mushrooms are abundant in ergosterol, which can be converted into vitamin D_2_ by ultraviolet (UV) illumination [27]. After ingestion, vitamin D_2_ undergoes a series of activation steps to give 1α,25-(OH)_2_ D_2_, which is believed to be equipotent to 1α,25-(OH)_2_D_3_ (calcitrol) in the prevention and cure of rickets and other vitamin D actions in the body through utilization of the same VDR-mediated regulation of gene expression. Thus vitamins D_3_ and D_2_ have been used interchangeably in supplements, but recent evidence suggests that vitamin D_2_ should not be regarded as equivalent to vitamin D_3_ [10,28,29].

In agreement with other studies, supplementation with vitamin D_2_ increased serum 25(OH)D_2_ but decreased serum 25(OH)D_3_ [30,31,32]. Little information is available on potential functional consequences of this metabolic response, but findings from the current study showing that muscle damage was heightened after eccentric exercise in the vitamin D_2_ supplemented NASCAR pit crew athletes indicate further research on functional outcomes is needed. High serum 25(OH)D_2_ is not a normal occurrence in humans except after the use of vitamin D_2_ supplements. Entry of vitamin D_2_ into the total body pool of vitamin D dilutes the relative amount of vitamin D_3_, resulting in a gradual replacement within the total pool of 25(OH)D and 1α,25-(OH)_2_D. Early evidence suggested that vitamin D-related cytochrome P450 enzymes including CYP2R1 and CYP27B1 (vitamin D activation) and CYP24A1 (inactivation) could not discriminate between vitamins D_2_ and D_3_. More recent evidence indicates that the vitamin D-dependent intestinal form of the drug-metabolizing cytochrome P450 enzyme, CYP3A4 (vitamin D inactivation), may discriminate against vitamin D_2_ [10]. CYP3A4 breaks down 1α,25-(OH)_2_D_2_ at a significantly faster rate than 1α,25-(OH)_2_D_3_, suggesting that this nonspecific cytochrome P450 enzyme might limit vitamin D_2_ action in target cells where it is expressed [10]. Thus, one explanation for the discrimination against vitamin D_2_ could be the selective catabolism of vitamin D_2_ by nonspecific cytochrome P450 enzymes in the liver and intestine. Despite a 3800 IU daily dose of vitamin D_2_, the athletes in the current study experienced a relatively low serum elevation in 25(OH)D_2_ (~8 ng/mL). How this may influence levels of muscle damage after eccentric exercise has yet to be determined.

This is the first report in the literature that athletes experienced more EIMD when supplemented with high doses of vitamin D_2_ (3800 IU/day) for six weeks. Whether or not the post-exercise elevations in CK and myoglobin were due to the combined physiological influence of elevated 25(OH)D_2_ and decreased 25(OH)D_3_ remains to be determined. The NASCAR pit crew athletes were not vitamin D deficient, with only one in each group below a serum 25(OH)D level of 30 ng/mL, the minimal threshold deemed necessary to overcome vitamin D deficiency. Thus further research is warranted to confirm whether or not vitamin D_2_ supplementation amplifies EIMD in athletes who are vitamin D deficient, especially when compared with vitamin D_3_ supplementation. Animal studies indicate that vitamin D_3_ supplementation promotes muscle regeneration and accelerated recovery of skeletal muscle strength after crush injury, with augmented cell proliferation and inhibition of apoptosis [33]. Thus vitamin D_3_ but perhaps not vitamin D_2_ supplementation in vitamin D deficient athletes, especially during periods of training with limited sun exposure, has the potential to improve recovery from intense exercise with an eccentric component.

In this study, muscle function test scores did not differ between the vitamin D_2_ and placebo groups after 6-weeks supplementation of pit crew athletes during their off season (December and January). A limitation of this study, given the heterogeneity of the athletes tested, was that group sample sizes were too low to detect significant differences unless large improvements in muscle function test scores were achieved. Data are limited, but other studies show varying performance responses to vitamin D_3_ supplementation, with results perhaps dependent on the degree of vitamin D deficiency in the athletic subjects [20,21]. In one study, Close *et al*. [34] showed no performance effect of 20,000 (*n* = 10) or 40,000 (*n* = 10) IU/week vitamin D_3_ *versus* placebo (*n* = 10) over a 12-week period in 30 club-level athletes, 57% of whom were vitamin D deficient. In another study by this research group, 8-weeks vitamin D_3_ supplementation (5000 IU/day) in vitamin D deficient athletes improved sprint and vertical jump performance compared to placebo, but subject number in each group was low (*n* = 5) [5]. Wyon *et al*. [21] showed that vitamin D_3_ supplementation (2000 IU/day) by vitamin D insufficient/deficient classical ballet dancers during the winter months improved isometric strength and vertical jump scores relative to controls. However, this study was non-randomized, and did not use placebo control methods.

## 5. Conclusions

In summary, the novel and unexpected finding of this randomized, double-blinded, placebo controlled study of 28 NASCAR pit crew athletes was that 6-weeks supplementation with vitamin D_2_ increased serum 25(OH)D_2_, decreased serum 25(OH)D_3_, and amplified EIMD. Vitamin D_2_ supplemented athletes experienced no change in muscle function test scores compared to the placebo control group. If these results are confirmed by others, underlying mechanisms explaining the negative effects of vitamin D_2_ supplementation on EIMD need to be explored, with a focus on VDR and cytochrome P450 enzyme interactions.

## References

[1] Holick M.F., Binkley N.C., Bischoff-Ferrari H.A., Gordon C.M., Hanley D.A., Heaney R.P., Murad M.H., Weaver C.M. (2011). Evaluation, treatment, and prevention of vitamin D deficiency: An Endocrine Society clinical practice guideline. J. Clin. Endocrinol. Metab..

[2] Bischoff-Ferrari H.A. (2012). Relevance of vitamin D in muscle health. Rev. Endocrine Metab. Dis..

[3] Ginde A.A., Wolfe P., Camargo C.A., Schwartz R.S. (2012). Defining vitamin D status by secondary hyperparathyroidism in the U.S. population. J. Endocrinol. Investig..

[4] Cannell J.J., Hollis B.W., Sorenson M.B., Taft T.N., Anderson J.J. (2009). Athletic performance and vitamin D. Med. Sci. Sports Exerc..

[5] Close G.L., Russell J., Cobley J.N., Owens D.J., Wilson G., Gregson W., Fraser W.D., Morton J.P. (2013). Assessment of vitamin D concentration in non-supplemented professional athletes and healthy adults during the winter months in the UK: Implications for skeletal muscle function. J. Sports Sci..

[6] Constantini N.W., Arieli R., Chodick G., Dubnov-Raz G. (2010). High prevalence of vitamin D insufficiency in athletes and dancers. Clin. J. Sports Med..

[7] Halliday T.M., Peterson N.J., Thomas J.J., Kleppinger K., Hollis B.W., Larson-Meyer D.E. (2011). Vitamin D status relative to diet, lifestyle, injury, and illness in college athletes. Med. Sci. Sports Exerc..

[8] Magee P.J., Pourshahidi L.K., Wallace J. M.W., Cleary J., Conway J., Harney E., Madigan S.M. (2013). Vitamin D status and supplementation in elite Irish athletes. Int. J. Sport Nutr. Exerc. Metab..

[9] Gordon P.L., Sakkas G.K., Doyle J.W., Shubert T., Johansen K.L. (2007). Relationship between vitamin D and muscle size and strength in patients on hemodialysis. J. Renal Nutr..

[10] Jones G. (2013). Extrarenal vitamin D activation and interactions between vitamin D_2_, vitamin D_3_, and vitamin D analogs. Ann. Rev. Nutr..

[11] Sato Y., Iwamoto J., Kanoko T., Satoh K. (2005). Low-dose vitamin D prevents muscular atrophy and reduces falls and hip fractures in women after stroke: A randomized controlled trial. Cerebrovasc. Dis..

[12] Annweiler C., Schott A.M., Berrut G., Fantino B., Beauchet O. (2009). Vitamin D-related changes in physical performance: A systematic review. J. Nutr. Health Aging.

[13] Ardestani A., Parker B., Mathur S., Clarkson P., Pescatello L.S., Hoffman H.J., Polk D.M., Thompson P.D. (2011). Relation of vitamin D level to maximal oxygen uptake in adults. Am. J. Cardiol..

[14] Muir S.W., Montero-Odasso M. (2011). Effect of vitamin D supplementation on muscle strength, gait and balance in older adults: A systematic review and meta-analysis. J. Am. Geriatr. Soc..

[15] Stockton K.A., Mengersen K., Paratz J.D., Kandiah D., Bennell K.L. (2011). Effect of vitamin D supplementation on muscle strength: A systematic review and meta-analysis. Osteoporos. Int..

[16] Toffanello E.D., Perissinotto E., Sergi G., Zambon S., Musacchio E., Maggi S., Coin A., Sartori L., Corti M.C., Baggio G. (2012). Vitamin D and physical performance in elderly subjects: The Pro.V.V.A study. PLoS One.

[17] Barker T., Henriksen V.T., Martins T.B., Hill H.R., Kjeldsberg C.R., Schneider E.D., Dixon B.M., Weaver L.K. (2013). Higher serum 25-hydroxyvitamin D concentrations associate with a faster recovery of skeletal muscle strength after muscular injury. Nutrients.

[18] Grimaldi A.S., Parker B.A., Capizzi J.A., Clarkson P.M., Pescatello L.S., White M.C., Thompson P.D. (2013). 25(OH) Vitamin D is associated with greater muscle strength in healthy men and women. Med. Sci. Sports Exerc..

[19] Janssen H.C., Emmelot-Vonk M.H., Verhaar H.J., van der Schouw Y.T. (2013). Vitamin D and muscle function: Is there a threshold in the relation?. J. Am. Med. Dir. Assoc..

[20] Moran D.S., McClung J.P., Kohen T., Lieberman H.R. (2013). Vitamin D and physical performance. Sports Med..

[21] Wyon M.A., Koutedakis Y., Wolman R., Nevill A.M., Allen N. (2013). The influence of winter vitamin D supplementation on muscle function and injury occurrence in elite ballet dancers: A controlled study. J. Sci. Med. Sport.

[22] Choi M., Park H., Cho S., Lee M. (2013). Vitamin D_3_ supplementation modulates inflammatory responses from the muscle damage induced by high-intensity exercise in SD rats. Cytokine.

[23] Smith L.L., Brunetz M.H., Chenier T.C., McCammon M.R., Houmard J.A., Franklin M.E., Israel R.G. (1993). The effects of static and ballistic stretching on delayed onset muscle soreness and creatine kinase. Res. Q. Exerc. Sport.

[24] Shanely R.A., Nieman D.C., Knab A.M., Gillitt N.D., Meaney M.P., Jin F., Sha W., Cialdella-Kam L. (2013). Influence of vitamin D mushroom powder supplementation on exercise-induced muscle damage in vitamin D insufficient high school athletes. J. Sport Sci..

[25] Huang M., Winters D. (2011). Application of ultra-performance liquid chromatography/tandem mass spectrometry for the measurement of vitamin D in foods and nutritional supplements. J. AOAC Int..

[26] Calton L.J., Molloy B.J., Keevil B.G., Cooper D.P. (2009). The analysis of 25-hydroxyvitamin D in serum using semi-automated solid phase extraction and LC/MS/MS. Clin. Chem..

[27] Urbain P., Singler F., Ihorst G., Biesalski H.K., Bertz H. (2011). Bioavailability of vitamin D_2_ from UV-B-irradiated button mushrooms in healthy adults deficient in serum 25-hydroxyvitamin D: A randomized controlled trial. Eur. J. Clin. Nutr..

[28] Houghton L.A., Vieth R. (2006). The case against ergocalciferol (vitamin D_2_) as a vitamin supplement. Am. J. Clin. Nutr..

[29] Tripkovic L., Lambert H., Hart K., Smith C.P., Bucca G., Penson S., Chope G., Hyppönen E., Berry J., Vieth R. (2012). Comparison of vitamin D_2_ and vitamin D_3_ supplementation in raising serum 25-hydroxyvitamin D status: A systematic review and meta-analysis. Am. J. Clin. Nutr..

[30] Biancuzzo R.M., Clarke N., Reitz R.E., Travison T.G., Holick M.F. (2013). Serum concentrations of 1,25-dihydroxyvitamin D_2_ and 1,25-dihydroxyvitamin D_3_ in response to vitamin D_2_ and vitamin D_3_ supplementation. J. Clin. Endocrinol. Metab..

[31] Logan V.F., Gray A.R., Peddie M.C., Harper M.J., Houghton L.A. (2013). Long-term vitamin D_3_ supplementation is more effective than vitamin D_2_ in maintaining serum 25-hydroxyvitamin D status over the winter months. Br. J. Nutr..

[32] Stephensen C.B., Zerofsky M., Burnett D.J., Lin Y.P., Hammock B.D., Hall L.M., McHugh T. (2012). Ergocalciferol from mushrooms or supplements consumed with a standard meal increases 25-hydroxyergocalciferol but decreases 25-hydroxycholecalciferol in the serum of healthy adults. J. Nutr..

[33] Stratos I., Li Z., Herlyn P., Rotter R., Behrendt A.K., Mittlmeier T., Vollmar B. (2013). Vitamin D increases cellular turnover and functionally restores the skeletal muscle after crush injury in rats. Am. J. Pathol..

[34] Close G.L., Leckey J., Patterson M., Bradley W., Owens D.J., Fraser W.D., Morton J.P. (2013). The effects of vitamin D_3_ supplementation on serum total 25[OH]D concentration and physical performance: A randomized dose-response study. Br. J. Sports Med..

